# The Pathogenesis of Necroptosis-Dependent Signaling Pathway in Cerebral Ischemic Disease

**DOI:** 10.1155/2018/6814393

**Published:** 2018-07-22

**Authors:** Yang Xu, Ji Zhang, Lingsong Ma, Shoucai Zhao, Shizun Li, Tingting Huang, Zhaohu Chu

**Affiliations:** ^1^Department of Neurology, First Affiliated Hospital of Wannan Medical College, No. 2, ZheShanXi Road, Wuhu 241001, China; ^2^Non-Coding RNA of Major Diseases Research Center, Central Laboratory, First Affiliated Hospital of Wannan Medical College, No. 2, ZheShanXi Road, Wuhu 241001, China

## Abstract

Necroptosis is the best-described form of regulated necrosis at present, which is widely recognized as a component of caspase-independent cell death mediated by the concerted action of receptor-interacting protein kinase 1 (RIPK1) and receptor-interacting protein kinase 3 (RIPK3). Mixed-lineage kinase domain-like (MLKL) was phosphorylated by RIPK3 at the threonine 357 and serine 358 residues and then formed tetramers and translocated onto the plasma membrane, which destabilizes plasma membrane integrity leading to cell swelling and membrane rupture. Necroptosis is downstream of the tumor necrosis factor (TNF) receptor family, and also interaction with NOD-like receptor pyrin 3 (NLRP3) induced inflammasome activation. Multiple inhibitors of RIPK1 and MLKL have been developed to block the cascade of signal pathways for procedural necrosis and represent potential leads for drug development. In this review, we highlight recent progress in the study of roles for necroptosis in cerebral ischemic disease and discuss how these modifications delicately control necroptosis.

## 1. Introduction

For a long time, necrosis was classified as nonprogrammed cell death as a response to extreme stress. The integrity of the plasma membrane was attacked by uncontrolled and accidental necrosis causing the collapse of the cell, though the nuclei keep substantially intact on the way [1]. However, in recent years, there is strong evidence confirming that part of necrosis also contained program control, therefore proposing the new concept as programmed necrosis or necroptosis. Apoptosis, autophagy, and necroptosis are all classified as programmed cell death (PCD) based on morphological and biochemical features [2, 3]. These phenomena have been observed in the ischemic stroke model [4–6]. Due to technical limitations, many studies considered necroptosis equated with apoptosis. Necroptosis is not induced by the caspase activation which is a typical requirement of the apoptotic pathway [7]. Wang et al. systematically exposed the classic signal pathway of necroptosis to further understand this form of cell death [8]. It was found that necroptotic cell death participates in a variety of cerebrovascular diseases. These mechanisms are reviewed in this paper, since they could be targets of new therapeutic approaches for these diseases.

## 2. Research Progress of the Signal Pathway of Programmed Necrosis

In the 1990s, researchers observed that caspase inhibition cannot fully block tumor necrosis factor- (TNF-) induced cell death but rather switches the cell fate to the necrotic death signal pathway similar to apoptosis [9, 10]. This is the first time that procedural necroptosis has been observed. In the activation of downstream necroptosis of the pathway, caspase-8 plays a critical regulatory role in the switch. Caspase-8 acts as an endogenous inhibitor of the necroptosis signal. It not only cleaves but also activates and initiates the execution phase of apoptosis. When FADD-caspase-8-FLIP complex functions are inhibited, the pathway of cell death switches from apoptosis to typical necroptosis features [11–14]. On the other side, when RIP3 kinase activity is inhibited, necroptosis may also lead to the activation of the FADD-RIPK1-RIPK3-caspase-8 complex to induce apoptosis [15]. TNF-*α* is the major trigger of necroptosis, which is capable of initiating RIPK1 kinase-dependent necroptosis as well as caspase-8-dependent apoptosis [16].

So what is the most classic feature of procedural necroptosis? TNF-*α*-induced necroptosis is mostly intensively investigated. TNF receptor 1 (TNFR1) ligation recruits complex I which contains TRADD, TRAF2, and cIAP1/2 [17]. Complex I transits into the cytosol activating death-inducing TNFR1 complex II via cylindromatosis (CYLD) [18]. In the necrotic signal pathway, receptor-interacting kinase 1 (RIPK1 or RIP1) was the first molecule recognized as the central components of the necroptotic machinery [19]. When FADD or caspase-8 is inactivated or absent, RIPK1 and TRIF include a RIP homotypic interaction motif (RHIM) domain that permits RIPK3 activation via RHIM-mediated interactions. RIPK3 kinase activity and RIPK3 RHIM domain are the requirement of necroptosis induction [20, 21]. RIPK1 and RIPK3 were phosphorylated, then formed a necrosome through their RHIM domains, and activate their kinase activities [22]. This RIPK1-RIPK3 interacts with mixed-lineage kinase domain-like (MLKL) phosphorylation [23], which consists of an N-terminal 4-helical-bundle domain (4HBD) linked by a brace region to a C-terminal pseudokinase domain [24]. MLKL was phosphorylated by RIP3 at the serine 358 and threonine 357 residues, which induces a conformational change into its active state [25], and then formed tetramers and translocated onto the plasma membrane, which injures cellular membrane integrity resulting in cell swelling and membrane rupture [26]. Downstream of the necrosome are two splice variants of phosphoglycerate mutase family member 5 (PGAM5), PGAM5S and PGAM5L. It was first reported to be a key substrate associated with the RIP1-RIP3-MLKL complex in necroptosis. PGAM5S and PGAM5L are both requirements of intrinsic necroptosis. The presence of the necrosis inhibitor necrosulfonamide (NSA) does not affect PGAM5L bound to the necrosome. However, the binding of PGAM5S is blocked by NSA. PGAM5S is normally located on the mitochondria and becomes related to the upstream necrosis-inducing complex probably through interactions with RIP3 resulting in the activation of PGAM5S by phosphorylation [27]. Furthermore, mitochondrial fragmentation caused by the mitochondrial phosphatase PGAM5S which recruited the mitochondrial fission factor dynamin-related protein 1 (Drp1) may upregulate ROS generation [27].

**Figure 1:**
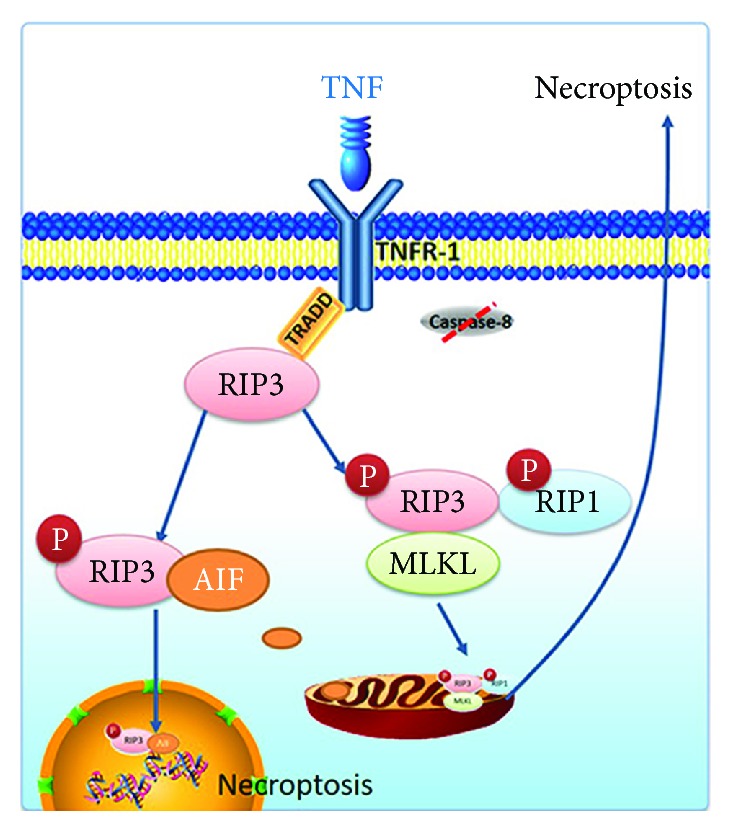
Two classic pathways of necroptosis. One of the classic pathways is RIP3 binding to RIP1 to form procedural necrosis complex II, which in turn binds to MLKL and mediates necroptosis of the mitochondrial pathway. The other is RIP1 binding to AIF, which translocated into the nucleus and mediates necroptosis of the nuclear pathway.

RIP3 is a nucleocytoplasmic shuttling protein whose nuclear distribution is temperature-sensitive. RIP3 possesses two classical unconventional nuclear localization signals (NLS, aa 442–472) and two classical nuclear export signals (NES) [28]. The above two localization signals and amino acid residue 452 within NLS participated in the necroptosis signal pathway [29]. Therefore, some studies began to explore the existence of the RIP3 nuclear pathway in addition to the classic mitochondrial pathway. Recent literature suggests that RIP3 binds to the apoptosis-inducing factor (AIF) nuclear pathway which is also involved in the process of procedural necrosis [14]. AIF is discharged from mitochondria and combines with RIP3 to form RIP3-AIF complexes. The new RIP3-AIF complex translocates into the nucleus leading to DNA degradation, and then the neurons are induced to suffer the necroptosis signal pathway (Figure 1).

## 3. Necroptosis in Cerebral Ischemia Disease

In the field of cerebral ischemia, the recognition of procedural necrosis can be traced back to 2005; Degterev et al. found that cerebral ischemia in mice in the absence of apoptotic signaling is contributing to the nonapoptotic death pathway like necroptosis. Necroptosis delayed mouse ischemic brain injury distinct from that of apoptosis [30]. Vieira et al. established oxygen-glucose deprivation (OGD) models in vitro and explored the mechanism underlying OGD-induced necroptosis in hippocampal neurons, and they found that although ischemic insults induced caspase-8 mRNA downregulation, they also induced RIP3 mRNA and protein level upregulation. The changes in RIP3 protein level were positively related to hippocampal neuronal death. Similar to RIP3, RIP1 protein levels were positively related to the activation of neuronal death. Both RIP1 and RIP3 contribute to necrotic cell death in hippocampal neurons challenged with OGD insult. Consistent with the classical procedural necroptosis cellular pathways, upregulation of RIP1-RIP3 expression and negative change of caspase-8 can afterwards be used to activate necroptotic signaling [31].

Global brain ischemia and reperfusion (I/R) injury acts as another manifestation of brain cell injury, in which the hippocampal CA1 layer is especially vulnerable [32, 33]. Yin et al. built rat 20 min global cerebral ischemia model to explore intracellular changes [5]. As a marker of necroptosis, RIP3 upregulated and transferred into the nucleus after cerebral ischemia and reperfusion injury. The RIP1-RIP3 complex plays crucial roles for TNF-induced necroptosis in the cell cytosol. ATP depletion is one of the results of the mitochondrial permeability transition pore (mPTP) leading to mitochondrial swelling. Fakharnia et al. presented additional arguments in the classical mitochondrial pathway. As a gatekeeper of mPTP, CypD, which mediated mPTP opening, may contribute to not only apoptosis but also necroptotic cell death in cerebral I/R injury and alleviated the levels of RIP1 and RIP3 [34]. However, the RIP3 function in the nucleus is not completely dependent on the RIP3-RIP1 complex. It needs future studies to examine other nuclear proteins that interacted with RIP3 [5]. Xu et al. continued to explore the role of RIP3 mechanisms in the nucleus. The interaction between activated RIP3 and AIF occurs in the cytoplasm after I/R injury. AIF and RIP3 translocation into the nucleus is critical to neuronal necroptosis, and AIF translocation into the nucleus may be RIP3-dependent [14]. As a key mediator, AIF links caspase-independent PCD with the necroptotic pathway.

Nerve cell necrosis was observed following the focal middle carotid artery occlusion/reperfusion (MCAO/R) ischemic stroke model. TNFR1 and RIP3 were positively expressed and significantly increased following the volume of cerebral infarction postreperfusion. Caspase inhibitor z-VAD-FMK (z-VAD) treatment markedly increased RIP3 expression during ischemia injury [35]. Compared with the classic procedural necrosis pathway, Lu et al. put forward the opposite view on the MCAO model, which is mainly concentrated in the downstream of RIP1-RIP3-MLKL. They found the mitochondria-enriched RIP1/RIP3 complex in necroptosis; nevertheless, PGAM5 had hardly any effect on RIP1-RIP3 recruitment. PGAM5 gene knockout in mice exacerbated necroptosis rather than reduced necroptosis. It causes abnormal mitochondrial accumulation and increases ROS generation [36].

In addition to phosphorylation modification, Miao et al. tested RIP3 S-nitrosylation in I/R paralleled with elevated phosphorylation. It means that phosphorylation and activation of RIP3 could be modulated by its S-nitrosylation triggered by NMDAR-dependent nNOS activation [37].

## 4. The Activation of the NLRP3 Inflammasome by a Mechanism Involving the RIP1-RIP3 Signaling Pathway

**Figure 2:**
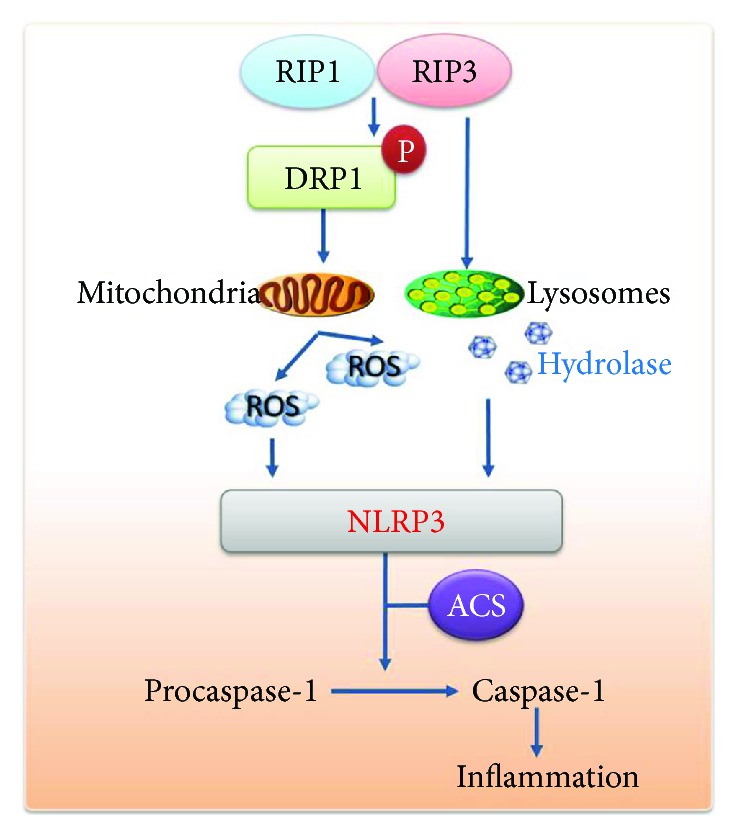
The activation of the NLRP3 inflammasome through the RIP1-RIP3 signaling pathway. The activation of RIP1-RIP3 damages the mitochondria by activating the DRP1. This results in excessive production of ROS and subsequent trigger activation of the NLRP3 inflammasome. RIP3 also destructs lysosomal membrane stability, leading to hydrolase release (such as cathepsin-B) and activation of NLRP3-mediated inflammatory factors.

Neuroinflammation is still the primary cause of morbidity and mortality in cerebral ischemia [38–40]. The NOD-like receptor pyrin 3 (NLRP3) inflammasome is considered an effective therapeutic target. The abnormalities of structure and expression of the NLRP3 inflammasome could affect the development or progression of ischemic stroke. After OGD, MCAO, or global cerebral ischemia injury, NLRP3 inflammasome and other proinflammatory cytokines were activated [41]. These cytokines could be the mediating molecules during postischemic inflammation and immune responses. Following the detection of cellular stress, NLRP3 was exposed to interact with the adaptor apoptosis-associated speck-like protein containing a CARD (ASC). After binding with NLRP3, ASC recruits procaspase-1 clustering which permits autocleavage and formation of the active caspase-1, which mediates the release of the mature, biologically active cytokines to engage in immune defense [42]. The downstream of NLRP3 was explored relatively and clearly in neuronal cell experiments, primarily associated with inflammatory factors. Caspase-1 and both IL-6 and IL-1*β* are involved in mouse primary cortical neurons' ischemic conditions [43, 44]. In particular, caspase-1 inhibitor treatment protected neurons in experimental stroke models through suppression of NLRP3 inflammasome activity [45]. These mechanisms have been confirmed in the cerebral ischemic disease model. However, we know very little about its upstream studies. So far, the mechanism that activates NLRP3 inflammasome in ischemia injury generalizations in two main models, lysosomal damage or ROS release, is mutually connected and associated with NLRP3 in ischemia injury (Figure 2). Although there are many pathways of ROS production after ischemia [46, 47], necroptosis is one of the ways.

As mentioned before, caspase-8 is a regulatory molecule. Kang found certain cells deficient in caspase-8 prompting which is mediated by RIP1 and RIP3. Caspase-8 deficiency dendritic cells (DCs) expressed to accentuated activation of the inflammasome through the functions of RIP1, RIP3, and MLKL [7]. And Gurung et al. considered caspase-8 an apical mediator of NLRP3 inflammasome priming [48]. MLKL, a key component downstream of necrosome components, is considered an executor of necroptosis. In MLKL-knockout mice, NLRP3 activation was inhibited, which means that the potential function of MLKL is the regulation of inflammasome activation [17]. Beyond their core role in necrosis, the necrosome components RIP1 and RIP3 have been proposed to be hyperresponsive to the induction of assembly, which were applied to the NLRP3 inflammasome. When inhibitory factors of necrosomes are blocked, RIP1-RIP3 could promote inflammasome activation spontaneously. Wang et al. discovered that the RIP1-RIP3 complex participates in RNA virus-induced NLRP3 activation through the GTPase DRP1 pathway. This promoted the mitochondrial damage leading to the production of ROS and stimulus NLRP3 [49].

The relationship between the procedural necrosis and inflammation is likely clearer, but this may not be the only signaling pathways that intersect. At least downstream studies, including the relationship between necroptosis and inflammation in cerebral infarction, indicate the direction of the study.

## 5. The Regulation of Necroptosis in the Cerebral Ischemic Model

Many studies focus on how to block the cascade of signal pathways for procedural necrosis. The most classic inhibitor is the small-molecule compound necrosulfonamide (NSA). NSA did not block interactions between necrosis-induced RIP1 and RIP3, but it blocks necroptosis downstream of RIP3 activation. NSA targets the N-terminal Cys86 residue MLKL which has a drawback that is specifically inhibiting necroptosis in multiple human cell lines. In human glioblastoma cells, NSA switches from necrosis to apoptosis in edelfosine-treated cells [50]. In a HeLa cell line in which caspase-8 was knocked down and RIP3 was expressed, necrosis induced by TNF-*α* plus Smac mimetic (no need for z-VAD) was efficiently blocked by NSA, while necrosis induced by either TNF-*α* or z-VAD was insensitive to NSA in a 3T3 cell line expressing mouse RIP3 [23]. NSA also significantly reduces BV6/MS275-induced cell death in acute myeloid leukemia (AML) cell lines [51].

In the field of cerebral ischemia, some of the inhibitors of programmed necrosis have been demonstrated in animal models. Degterev et al. reported that as an identified small-molecule inhibitor of necroptosis, necrostatin-1 (NEC-1) has been shown to ameliorate tissue damage in ischemic brain injury animal models [30]. NEC-1 has a selective primary cellular target responsible for the death domain receptor-associated adaptor kinase RIP1 activity [52, 53]. The crystal structures of the RIP1 kinase domain bound to NEC-1 and are caged in a hydrophobic pocket between the N- and C-lobes of the kinase domain. This structure stabilizes RIP1 in an inactive conformation. DAXX is a novel substrate of RIP3 in global cerebral ischemia and ischemia of the retinal cell animal model and is translocated from the nucleus to the cytoplasm in response to stress. Pretreatment with Nec-1 can block DAXX translocation from the nucleus to the cytoplasm, which resulted in the inactivation of DAXX [54–56]. NEC-1 not only inhibited the expression of RIP1 and prevented the upregulation and nuclear translocation of RIP3 but also decreased cathepsin-B release in the globe cerebral ischemic model. This suggests that there may be a signal transduction between programmed necrosis and autophagy [5]. The recent literature highlights the intricate interplay between necroptosis and autophagy. CA074-me and 3-methyladenine (3-MA), as autophagy inhibitors [46], were used to determine what is beneficial for global cerebral ischemia in the process of necroptosis signal pathways. CA074-me and 3-MA pretreatment greatly inhibited rat mortality rates and neuronal death. The mechanism of 3-MA is the inhibition of the nuclear translocation and colocalization of RIP3 and AIF, as it is significant for ischemic neuronal DNA degradation and necroptosis for the nuclear translocation of the RIP3-AIF complex [14]. In the stabilization of the lysosomal membrane, CA074-me has an indirect effect by maintaining energy balance and inhibiting RIP3 expression and nuclear translocation [57]. Besides this, CA074-me almost completely hampered the loss of mitochondrial membrane depolarization, phosphatidylserine (PS) translocation, and plasma membrane rupture [58].

Multitargeted kinase inhibitors, such as dabrafenib, vemurafenib, sorafenib, pazopanib, and ponatinib, are currently used for the treatment of cancer, which have later emerged as having antinecroptotic activity [59–61]. Among them, dabrafenib may block TNF-*α*-induced necroptosis as an effective high-affinity inhibitor of RIP3. Dabrafenib administered intraperitoneally after mouse cerebral ischemic injury markedly reduced infarct lesion size along with significantly attenuated upregulation of TNF-*α* [59].

## 6. Future Research about Necroptosis

Previous studies suggested that PGAM5 promotes necroptosis by associating with necrosome. However, the role of PGAM5 intrinsically is still controversial [62]. In response to multiple necroptotic stimuli, PGAM5 deficiency aggravated rather than mitigated necroptosis in brain ischemic reperfusion injury [36]. The loss of PGAM caused abnormal mitochondrial accumulation and increased ROS generation. In oxidative damage and necroptosis-dependent stroke, PGAM5 could drive pathology, and thus targeting PGAM5 may be of benefit.

The core of procedural necrosis is RIP1-RIP3 and its downstream signaling pathways. In addition to the above-mentioned inhibitors, the drugs used in the experiments and the clinical use of drugs have been developed. This is because the understanding of this pathway is still relatively limited. After the classic pathway was proposed, there are many studies that questioned the effect of RIP3 on the necrosis of the bypass signals, such as dynamin-related protein 1 and proteasome beta-4 subunit (PSMB4) [63, 64]. These need to be systematically assessed. Furthermore, the most critical problem is that for a long time, both necrotic and apoptotic cells dominated the theory of neuronal death in the penumbra zone. The clarification of the relationship between procedural necroptosis and necrosis and autophagy still needs a lot of work to do. This cross-channel signal path may be complexity over imagination.
